# Anxiety, depression, working from home and health-related behaviours during COVID-19: Structural equation modelling and serial mediation of associations with angina, heart attacks and stroke

**DOI:** 10.1177/13591053241241412

**Published:** 2024-03-28

**Authors:** Bárbara Lopes, Caroline Kamau-Mitchell

**Affiliations:** 1 Center for Research in Neuropsychology and Cognitive Behavioural Intervention (CINEICC), Faculdade de Psicologia e de Ciãncias da Educação, Universidade de Coimbra, Portugal; 2 Centre for Medical Humanities, and Birkbeck Business School, Birkbeck, University of London, UK

**Keywords:** alcohol consumption, anxiety, cardiovascular diseases, depression, early COVID-19, health behaviours, smoking, sugar consumption, working from home

## Abstract

Based on the vulnerability-stress model and coping theory, this study of 1920 people in Scotland investigated how sex, age, occupational factors, anxiety, depression and maladaptive coping behaviours are associated with cardiovascular health. Structural equation modelling and serial Sobel mediation tests were conducted. Anxiety was associated with past arrhythmia, whereas depression was associated with past heart attacks, stroke and angina. Females reported more anxiety, past arrhythmia, confectionary and alcohol consumption, whereas males had more heart attacks. Confectionary consumption was associated with past arrhythmia, and alcohol consumption was associated with past heart attacks. Being older was associated with depression, past stroke, arrhythmia and alcohol consumption. Being younger was associated with anxiety and smoking. Depression and smoking mediated the relationship between type of working and cardiovascular health history, potentially because of socioeconomic factors. Clinicians can use these results to advise clients about cardiovascular risks associated with anxiety, depression, demographics and health-related coping behaviours.

## Introduction

Anxiety and depression are the most common mental health problems worldwide and researchers argue that they increase the risk of cardiovascular diseases (CVDs) such as stroke and heart attacks (Cohen et al., 2015), and deaths from CVDs (Celano et al., 2016; Harshfield et al., 2020; Reiner et al., 2020; Tully et al., 2016). CVDs harm the mental health and quality of life of patients who survive by causing physical and/or neurological deficits or disabilities, and they are major causes of death in countries such as Scotland where there were 3932 deaths from cardiovascular diseases alone in 2020 (Public Health Scotland, 2021). In light of the vulnerability × stress model (Zubin and Spring, 1977) and coping theory (Cohen and Lazarus, 1979), this study investigated the relationships between primary risk factors (e.g., sex, age, occupational factors) and secondary risk factors (e.g. symptoms of depression, anxiety and maladaptive coping behaviours such as smoking, drinking alcohol and a sugary diet) during the COVID-19 pandemic with cardiovascular health (indicated by CVD history). This extends previous literature (e.g., Reiner et al., 2020) by considering both types of risk factors holistically and by distinguishing between cardiovascular diseases (e.g., stroke, angina and heart attacks) and arrhythmia.

### Cardiovascular diseases and vulnerability × stress

Cardiovascular diseases develop because of problems with blood vessels supplying oxygen-rich blood to the brain (Abrignani et al., 2014), but there is growing recognition that mood states contribute to these biological processes (Karlsen et al., 2021a, 2021b). Anxiety and depressive mood are associated with cortisol, a stress hormone which nearly triples the risk of cardiovascular diseases (Manenschijn et al., 2013). They are also associated with serotonergic neurotransmission (Reimold et al., 2011), which is notable because cortisol serotonergic action is associated with cardiovascular functioning (Monassier and Morateux, 2019).

Although evidence is clear that anxiety and depression are associated with cardiovascular diseases such as stroke and heart attacks (Celano et al., 2016; Harshfield et al., 2020; Reiner et al., 2020; Tully et al., 2016), evidence about arrhythmia is unclear. Arrhythmias are abnormalities in heart rhythm which involve beating too quickly (tachycardia), too slowly (bradycardia) or irregularly. They are not defined as CVDs but they are associated with higher risks of stroke and heart attacks (Bordignon et al., 2012), and are also important to consider within clinical practice because arrhythmia can be a symptom of anxiety disorders such as panic disorder and generalised anxiety disorder (Abrignani et al., 2014; see the American Psychiatric Association’s (APA), 2013). This study will add a new approach to the literature by examining both abnormal heart rhythm and CVDs (angina, stroke and heart attacks).

Drawing from the vulnerability × stress model by Zubin and Spring (1977), this study proposed that demographics such as sex and age (which are biological characteristics) and occupational factors (as situational antecedents) are vulnerabilities associated with poor mental health and CVDs whose implications were made complex by the COVID-19 pandemic, which was the context for this study. For example, whereas older age is associated with higher cardiovascular risks (e.g., Rodgers et al., 2019), as is being male (Karlsen et al., 2021a; Lee et al., 2022), mental correlates of CVDs and their prevalence during the COVID-19 pandemic might have made these associations more complicated. For example, if females tended to suffer more anxiety than males, and younger people tended to experience more anxiety than older people during the pandemic (Gao et al., 2019; Hubbard et al., 2021; Reiner et al., 2020), it suggests that these groups could also be at risk of CVDs because of secondary risk factors (Ferreira et al., 2019; Karlsen et al., 2021b; Reiner et al., 2020). Therefore, both males and females and younger and older people could be at risk of poor cardiovascular health through different pathways. Added to this, recent studies suggested that working from home during the COVID-19 pandemic was a risk factor for the onset of depression and anxiety due to workers feeling trapped at home and burdened by both childcare and work-related responsibilities (Burn et al., 2022; Mendonça et al., 2022). This study proposed that, together with biological vulnerabilities, working from home was likely associated with poor mental health in terms of anxiety and depression which, in turn, were likely associated with cardiovascular risk as previous studies suggest (e.g., Ferreira et al., 2019) as well as poor health-related coping behaviours (Ferreira et al., 2019).

According to coping theory by Cohen and Lazarus (1979), certain behaviours raise or lower the risk of diseases or illnesses. Certain poor health-related coping behaviours (e.g., smoking, alcohol consumption, poor diet habits) are associated with higher risks of CVDs (Ferreira et al., 2019) because people use such behaviours to cope with distressing emotions and negative mood (e.g., depression and anxiety) during stressful periods (Christofaro et al., 2022; Ferreira et al., 2019; Grases et al., 2019; Stanton et al., 2020; Tully et al., 2016). This suggests that some people may have used certain behaviours to cope during the COVID-19 pandemic because it was a time when there were high levels of distress within the general population (Dicken et al., 2022; Naughton et al., 2021; Stanton et al., 2020). Indeed, studies found an increase in alcohol consumption (Naughton et al., 2021), smoking and sweet consumption (Dicken et al., 2022) compared to before the pandemic (Schäfer et al., 2022) and these trends were stronger among younger people and females compared to older people and males (Jackson et al., 2022; Naughton et al., 2021),illustrating why the COVID-19 pandemic likely complicated the implications of primary risk factors in terms of vulnerability. Indeed, females and younger people might have been more vulnerable because of stressors such as childcare responsibilities, or lack of social support and social isolation due to schools/universities closing (Stanton et al., 2020; Thibaut and van Wijngaarden-Cremers, 2020). Therefore, understanding CVD risk likely requires understanding not just the behaviours themselves but also their correlates.

Although both anxiety and depression have been associated with CVD risk and CVD-related deaths (Harshfield et al., 2020; Karlsen et al., 2021a, 2021b; Reiner et al., 2020), meta-analyses which found evidence of a relationship between anxiety and CVDs (Batelaan et al., 2016; Roest et al., 2010) have been criticised because they failed to control for depression (e.g. Roest et al., 2010; Tully, 2017). Some studies found evidence in favour of anxiety as the precursor of depression (Bruce et al., 2016; Tully, 2017), whereas other researchers suggested that depression, and not anxiety, is the main risk factor for CVDs and that anxiety is associated with CVDs only when depression is also present (Karlsen et al., 2021b). These studies therefore show that new research is needed to offer clarification about the relationships among anxiety, depression, primary/secondary risk factors for CVDs and CVDs. Anxiety and depression are often comorbid therefore this study sought to advance the literature by exploring whether anxiety is associated with CVDs through depression as a mediator.

### Hypothesis

According to past literature about the impact of anxiety on CVDs (Karlsen et al.,2021a, 2021b) it is hypothesised that, during the early COVID-19 pandemic in Scotland, age and sex were associated with anxiety (Reiner et al., 2020), that working from home was associated with anxiety (Burn et al., 2022), and that these in turn should have direct and indirect associations with cardiovascular health (indicated by a history of abnormal heart rhythm and cardiovascular diseases: angina, heart attacks and stroke) through poor diet habits (defined as consumption of sweets and sugary soft drinks) and through maladaptive coping factors known to increase the risk of CVDs (alcohol use and smoking) as well as depression.

## Method

### Participants and design

This study examined data from 1920 participants aged over 16 years who agreed to take part in a telephone survey in response to a letter from ScotCen Social Research (2021) which they sent to a random sample of 11,000 addresses in Scotland. In this study, variables of interest were potential primary risk factors for CVDs (age, sex and occupational factors), and secondary risk factors which we explored as mediators (anxiety, depression, diet, smoking and alcohol consumption). The outcome variables were respondents’ history of CVDs (namely, heart attacks, stroke and angina), and abnormal heart rhythm as indicators of their cardiovascular health. Since abnormal heart rhythm is considered to be a predisposing factor in CVDs (Bordignon et al., 2012), this will be analysed as having a unidirectional relationship with CVDs.

### Procedure

The Scottish Health Survey was carried out by ScotCen Social Research (2021) on behalf of the Scottish Government Health Directorates and NHS Health Scotland. Participants responded to the letter from ScotCen by opting-in to the survey. The interviewers telephoned the participants and asked them to provide verbal informed consent. The interviewers asked the participants to respond to a series of questions about their household, health, lifestyle and social circumstances. The interviewers conducted a survey of all willing adults within each household that opted-in. Participants received a £10 shopping voucher. Data collection occurred between August/September 2020. The response rate was good, in 70.1% of the responding households, all those aged 16 and above were interviewed (10.4% of eligible households).

### Ethical statement

Ethical approval for the 2020 telephone survey was obtained from the Health and Care Research Ethics Committee for Wales (REC reference number: 17/WA/0371). The dataset generated by the survey and analysed during the current study is available in the UK Data Service repository (see ScotCen Social Research, 2021). No identifiable information was collected, and verbal informed consent was provided by participants, based on their reading of the study’s information leaflet sent with the letter.

### Measures and questions

We examined participants’ responses to questions about their age; sex (Male = 0; Female = 1); diet (frequency of consuming confectionary and sugary soft drinks using a Likert Response Scale 1 = less often or never to 9 = six or more times a day); change in smoking and in alcohol consumption since lockdown (using a Likert Response scale: 1 = yes, decreased to 5 = yes, increased); cardiovascular health indicated by whether they have ever had a heart attack, stroke, angina and/or abnormal heart rhythm in the past (0 = No; 1 = Yes for each); type of working during the lockdown period (dummy coded into the binary dummy variables of working from home, working outside home, and both, where for each 0 = does not apply, 1 = applies); anxiety and depression. We examined the answers provided in the Clinical Interview Schedule-Revised (CIS-R) that measured anxiety (11 items) based on Lewis et al. (1992). Example questions asked about whether they felt anxious or nervous in the past month, whether they had physical symptoms such as a racing heart, dizziness, butterflies in the stomach, difficulty breathing and the duration of the anxiety. Depression was examined with the CIS-R for depression (eight items), which included questions about frequency of depressive feelings, their duration, and symptoms such as the inability to enjoy or take interest in things. Total scores for depression and anxiety are calculated by adding up the CIS-R items of depression and anxiety. Participants scoring above 12 on the CIS-R in the depression section were regarded as suffering from clinical depression whereas those scoring above 12 in the anxiety section were regarded as suffering from clinical anxiety (Brugha et al., 1999). Previous research suggested that the CIS-R is a valid measure, showing moderate concordance with ICD-10 diagnoses of depression and anxiety (Jordanova et al., 2004). Cronbach alphas suggested that the CIS-R anxiety and depression were reliable with α = 0.74 and α = 0.80, respectively. Full information about all the questions in the survey can be accessed via ScotCen Social Research (2021).

### Statistical analyses

SPSS and AMOS version 22 were used to conduct the analyses. Spearman Rho’s correlations were performed to analyse relationships between the variables. A Structural Equation Model was conducted in AMOS to test relationships among age, sex, working from home, outside home and both, anxiety, depression, frequency of eating confectionary and drinking sugary soft drinks, change in smoking and in drinking alcohol between lockdown and data collection and CVDs. Sobel tests were conducted to test for the significance of mediations, including serial mediations that analyse a causal chain linking several mediations (Hayes, 2022).

## Results

### Normality checks

Skewness and Kurtosis values for the variables were acceptable for Structural Equation Modelling (skewness −3 to +3; kurtosis −7 to +7) (see Table 1) (Brown, 2006).

**Table 1.** Descriptives for age, gender, anxiety, depression, frequency of eating confectionary (sweets and chocolate), frequency of drinking sugary soft drinks, type of working, change in smoking and in drinking alcohol between lockdown on the 23rd of March 2020 and August/September 2020.

Variables	*M*	SD		Minimum	Maximum	Skewness	Kurtosis
Age	57.53	17.21		16	90	−0.49	−0.55
Anxiety	8.50	4.26		2	24	1.43	0.54
Depression	5.57	4.16		1	19	1.08	−0.16
Frequency of eating confectionary	3.88	1.83		1	9	0.07	−0.86
Frequency of drinking sugary soft drinks	1.73	1.56		1	9	2.27	4.23
Change in alcohol consumption	2.59	0.73		1	3	−1.44	0.39
Change in smoking	3.76	0.93		3	5	0.49	−1.67
Sex	*Male* *n* = 813 (42.3%)	*Female* *n* = 1107 (57.7%)				−0.31	−1.90
Type of working	*Working from home* *n* = 300 (37.8%)	*Working outside home* *n* = 351 (44.2%)	*Both* *n* = 143 (18%)			0.32	−1.04

### Descriptive statistics

See Table 1 for the descriptives. Out of 1920 participants 218 reported past abnormal heart rhythm, 83 reported past angina, 83 reported past stroke and 80 reported past heart attacks. It is important to note that 24.5% (*n* = 471) of individuals met the criteria for clinical anxiety whereas 12.5% (*n* = 239) individuals met the criteria for clinical depression in this sample, thus suggesting a higher prevalence of clinical anxiety in comparison to the study by Hubbard et al. (2021) (13.8%).

### Spearman’s Rho correlations

One-tailed spearman’s rho correlations can be seen in Table 2.

**Table 2.** One-tailed Spearman Rho’s correlations.

	1	2	3	4	5	6	7	8	9	10	11
1. Age		−0.03	−0.18**	−0.13**	−0.24**	−0.15*	0.12**	0.17**	0.13**	0.16**	0.17**
2. Depression			0.28**	0.00	0.05**	0.12*	0.12*	0.05*	0.06**	0.05*	0.06**
3. Anxiety				0.08**	0.08**	0.07	0.08**	−0.01	0.03	−0.03	−0.03
4. Frequency of eating confectionary					0.15**	0.09	−0.01	−0.03	0.08**	−0.03	0.07**
5. Frequency of drinking sugary soft drinks						0.09	−0.07**	−0.03	0.08**	0.05*	0.05**
6. Change in smoking							0.14*	0.01	0.08	0.15*	0.03
7. Change in drinking alcohol								0.05*	−0.01	0.04	0.00
8. Heart attack									0.18**	0.11**	0.34**
9. Abnormal heart rhythm										0.11**	0.16**
10. Stroke											0.16**
11. Angina											

* *p* < 0.05. **p < 0.005.

### Structural Equation Model

**Figure 1.**
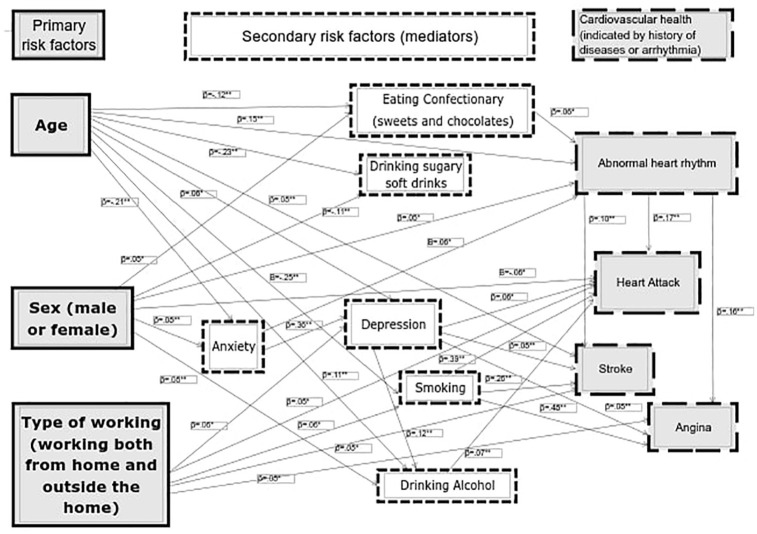
Structural Equation Model showing the primary and secondary risk factors (mediators) for cardiovascular health (arrhythmia, angina, stroke and angina). Primary risk factors are age, sex and type of working, and secondary risk factors are diet, anxiety, depression, smoking and alcohol use. Non-statistically significant pathways are not included. *p < 0.05. **p < 0.005.

Structural Equation Modelling (SEM) was conducted in AMOS to analyse the relationships among the variables in the study. Through different exploratory models, the model with the best fit comprised of independent variables (age, sex, working from home, outside home and both as dummy-coded variables) whose relationships with past abnormal heart rhythm, heart attacks, angina and stroke were mediated by anxiety, depression, poor diet and change in smoking and in alcohol consumption between lockdown and data collection. Model fit indices were good with a Root Mean Square of Approximation (RMSEA) = 0.050; Comparative Fit index (CFI) = 0.90, Bentler-Bonnett of Normed Fit Index (NFI) = 0.86 and CMIN/DF = 5.098 (Kline, 2005). Chi-Square (47, 1920) = 1092.020, *p* < 0.001 (see Figure 1).

Results for the different pathways are presented in Table 3. Results for the Sobel tests are presented in Table 4. As it can be observed, all the mediation pathways, including serial mediations, presented a statistically significant Sobel test statistic.

**Table 3.** Direct and indirect pathways between the main predictors (sex, age and working from home, outside home and both), the mediators (anxiety, depression, eating confectionary, drinking sugary soft drinks, alcohol consumption and smoking) and cardiovascular health (abnormal heart rhythm, stroke, heart attack and angina).

Direct pathways	β	SE	95% CI	*p*
Age → anxiety	−0.21	0.005	−0.020, −0.021	**<0.001**
Age → depression	0.06	0.005	0.050, 0.069	**0.009**
Age → frequency of eating confectionary	−0.12	0.002	−0.123, −0.116	**<0.001**
Age → frequency of drinking sugary soft drinks	−0.23	0.002	−0.233, −0.226	**<0.001**
Age→ smoking	−0.25	0.003	−0.255, −0.244	**<0.001**
Age → alcohol consumption	0.11	0.001	0.108, 0.111	**<0.001**
Age → abnormal heart rhythm	0.15	0.001	0.148, 0.151	**<0.001**
Age → stroke	0.08	0.001	0.078, 0.081	**0.002**
Age → heart attack	0.05	0.001	0.048, 0.052	0.082
Sex → anxiety	0.08	0.191	−0.294, 0.454	**<0.001**
Sex → frequency of eating confectionary	0.05	0.084	−0.114, 0.214	**0.016**
Sex →frequency of drinking sugary soft drinks	−0.11	0.070	−0.247, 0.027	**<0.001**
Sex → alcohol consumption	0.08	0.038	0.005, 0.154	**0.002**
Sex → smoking	0.07	0.083	−0.092, 0.232	0.058
Sex → abnormal heart rhythm	0.05	0.015	0.020, 0.079	**0.019**
Sex → heart attack	−0.06	0.010	−0.079, −0.040	**0.008**
Working from home → anxiety	0.02	0.260	0.598, 1.64	0.38
Working from home → depression	−0.01	0.339	−0.448, 0.969	0.50
Working from home → smoking	−0.04	0.176	−0.181, 0.563	0.35
Working from home→ heart attack	−0.01	0.021	−0.079, −0.011	0.45
Working from home → angina	−0.02	0.023	−0.066, 0.003	0.63
Working from home → stroke	−0.02	0.020	−0.081, −0.012	0.42
Working outside home → anxiety	−0.03	0.24	−0.022, 0.963	0.15
Working outside home → depression	−0.08	0.23	−0.712, 0.250	0.096
Working outside home → smoking	−0.04	0.17	−0.311, 0.289	0.14
Working outside home → heart attack	−0.02	0.01	−0.079, −0.011	0.11
Working outside home → angina	−0.02	0.01	−0.066, 0.003	0.14
Working outside home → stroke	−0.01	0.01	−0.081, −0.012	0.67
Working both outside and from home → anxiety	−0.03	0.04	−0.913, 0.539	0.55
Working both outside and from home → depression	0.06	0.25	−0.829, 0.196	**0.007**
Working both outside and from home → smoking	0.06	0.34	−0.500, 0.900	**0.008**
Working both outside and from home → heart attack	0.05	0.02	−0.058, −0.009	**0.011**
Working both outside and from home → angina	0.08	0.02	−0.062, −0.015	**0.006**
Working both outside and from home → stroke	0.05	0.02	−0.045, 0.002	**0.015**
Indirect pathways	β	SE	95% CI	*p*
Anxiety → abnormal heart rhythm	0.06	0.002	0.056, 0.063	**0.018**
Abnormal heart rhythm → heart attack	0.17	0.016	0.138, 0.201	**<0.001**
Abnormal heart rhythm → angina	0.16	0.017	0.126, 0.193	**<0.001**
Abnormal heart rhythm → stroke	0.10	0.016	0.068, 0.131	**<0.001**
Anxiety → depression	0.36	0.021	0.318, 0.401	**<0.001**
Depression → alcohol consumption	0.12	0.005	0.110, 0.129	**<0.001**
Alcohol consumption → heart attack	0.07	0.007	0.056, 0.083	**0.003**
Alcohol consumption → abnormal heart rhythm	0.03	0.011	0.008, 0.051	0.33
Depression → heart attack	0.06	0.001	0.058, 0.061	**0.044**
Depression → angina	0.08	0.002	0.076, 0.083	**0.022**
Depression → stroke	0.08	0.001	0.078, 0.081	**0.003**
Frequency of consuming confectionary → abnormal heart rhythm	0.06	0.004	0.052, 0.067	**0.005**
Frequency of drinking sugary soft drinks → abnormal heart rhythm	0.03	0.005	0.020, 0.039	0.40
Smoking → abnormal heart rhythm	0.06	0.015	0.030, 0.089	0.25
Smoking → heart attack	0.39	0.009	0.372, 0.407	**<0.001**
Smoking → angina	0.48	0.009	0.462, 0.497	**<0.001**
Smoking → stroke	0.26	0.008	0.244, 0.275	**<0.001**

Statistically significant effects are in bold.

**Table 4.** Sobel tests for the mediation pathways in the structural equation model.

Mediations	Sobel statistic	*p*
Age → frequency of consuming confectionary (sweets and chocolates) → abnormal heart rhythm	19.98	**<0.001**
Age → anxiety → abnormal heart rhythm	13.78	**<0.001**
Age → depression → heart attack	27.74	**<0.001**
Age → depression → angina	29.14	**<0.001**
Age → depression → stroke	29.14	**<0.001**
Age →alcohol consumption → heart attack	9.97	**<0.001**
Age → smoking → heart attack	9.93	**<0.001**
Age → smoking → angina	9.92	**<0.001**
Age → smoking → stroke	9.90	**<0.001**
Sex → frequency of eating confectionary → abnormal heart rhythm	5.40	**0.003**
Sex → anxiety → abnormal heart rhythm	10.98	**<0.001**
Anxiety → depression → heart attack	10.07	**<0.001**
Anxiety → depression → angina	16.36	**<0.001**
Anxiety → depression → stroke	16.36	**<0.001**
Sex → alcohol consumption → heart attack	1.98	**0.04**
Type of working: working both outside and from home → smoking → heart attack	1.98	**0.04**
Type of working: working both outside and from home → smoking → angina	5.11	**0.002**
Type of working: working both outside and from home → smoking → stroke	5.11	**0.002**
Type of working: working both outside and from home → depression → heart attack	3.33	**0.008**
Type of working: working both outside and from home → depression → angina	3.33	**0.008**
Type of working: working both outside and from home → depression → stroke	3.34	**0.007**
Serial mediations	Sobel statistic	*p*
Age → anxiety → depression → heart attack	8.69	**<0.001**
Age → anxiety → depression → stroke	8.69	**<0.001**
Age → anxiety → depression → angina	9.47	**<0.001**
Age → frequency of consuming confectionary (sweets and chocolates) → abnormal heart rhythm → heart attack	8.38	**<0.001**
Age → frequency of consuming confectionary(sweets and chocolates) → abnormal heart rhythm → stroke	5.62	**<0.001**
Age → frequency of consuming confectionary(sweets and chocolates) → abnormal heart rhythm → angina	8.64	**<0.001**
Age → anxiety → abnormal heart rhythm → heart attack	7.54	**<0.001**
Age → anxiety → abnormal heart rhythm → stroke	5.33	**<0.001**
Age → anxiety → abnormal heart rhythm → angina	7.70	**<0.001**
Sex → anxiety → depression → heart attack	2.46	**0.013**
Sex → anxiety → depression → stroke	2.46	**0.013**
Sex → anxiety → depression → angina	2.59	**0.009**
Sex → anxiety → abnormal heart rhythm → heart attack	2.96	**0.003**
Sex> anxiety → abnormal heart rhythm → stroke	2.76	**0.006**
Sex → anxiety → abnormal heart rhythm → angina	3.30	**<0.001**
Sex → frequency of consuming confectionary (sweets and chocolates) → abnormal heart rhythm → heart attack	2.33	**0.002**
Sex → frequency of consuming confectionary (sweets and chocolates) → abnormal heart rhythm → stroke	2.24	**0.025**
Sex → frequency of consuming confectionary (sweets and chocolates) → abnormal heart rhythm → angina	2.74	**0.006**
Type of working: working both outside and from home → depression→ alcohol consumption → heart attack	3.95	**<0.001**

Statistically significant effects are in bold.

## Discussion

This study extends knowledge about the associations among anxiety, depression and cardiovascular health, demonstrating the implications of sex, age and occupational factors such as working from home during the COVID-19 pandemic. The pandemic was a time of heightened stress and vulnerability among some populations (Wright et al., 2022) and our results showed, as previous literature suggested (Karlsen et al., 2021a, 2021b), that anxiety during the pandemic had an indirect association with poor cardiovascular health (indicated by CVD history) through depression. Moreover, as previous literature suggested (Karlsen et al., 2021a, 2021b), depression was associated with a history of angina, heart attacks and stroke. This extends a recent review of evidence showing that mental illness and cardiovascular health are associated (Lambert et al., 2022) by contributing new evidence about anxiety and depression.

The results advanced previous literature on the topic by showing that sex and age were important factors to consider in understanding the association between mental health and CVDs. Results showed that while males were more likely to have suffered heart attacks, as previous literature suggested (Karlsen et al., 2021a, 2021b), females were also at risk of poor cardiovascular health (Gao et al., 2019) through certain mediators. Females were more likely to have suffered abnormal heart rhythm, which was associated with CVDs, and females also had more anxiety, which was associated with depression and, in turn, CVDs. Females also showed poorer diet habits, such as consuming more sugary foods and alcohol than males, with both maladaptive coping mechanisms linked to abnormal heart rhythm and heart attacks (Ferreira et al., 2019).

Concerning the role of age, results showed that being older was associated with poorer cardiovascular health (indicated by a history of stroke and abnormal heart rhythm), more alcohol consumption and depression, both of which are risk factors for CVDs (Karlsen et al., 2021a, 2021b; Rodgers et al., 2019). Similar to the study by Reiner et al. (2020), the current results showed that younger people had more anxiety, which in itself is a risk factor for CVDs (Tully, 2017), in comparison to older people. Younger people also had a poorer diet, which was associated with a history of abnormal heart rhythm, and they smoked more during the early COVID-19 pandemic, with smoking associated with a history of CVDs. This suggested that the mechanisms behind cardiovascular health differed according to age. Although there was a direct relationship between being older and having a history of CVDs, younger people may also have poor cardiovascular health because of indirect reasons such as consuming more confectionary, being more anxious and smoking more, all of which are major risk factors for CVDs (Ferreira et al., 2019).

Our results also added to previous literature (e.g., Karlsen et al., 2021a, 2021b) by examining the relationships between type of working, history of CVDs and risk factors such as smoking, drinking alcohol, having a poor diet and mental health during the early COVID-19 pandemic. Contrary to our predictions, the SEM results showed that working both at home and outside the home during the pandemic was associated with depression and smoking, which in turn were associated with poorer cardiovascular health. This might be because population surveys (Office for National Statistics, 2020a, 2020b) found that people who had the opportunity to be solely working from home before and during the pandemic tended to be in better paid, professional jobs and were more educated. Such populations are associated with lower cardiovascular disease risks (Carter et al., 2019). After the pandemic started in April 2020, the Office for National Statistics (2020b) found that over 40% of employed people in Scotland engaged in working from home within the preceding week (a similar percentage to the UK average). The Office for National Statistics (2020b) survey found that over 60% of managers, directors, people in professional occupations and associate professional occupations engaged in working from home, compared to less than 20% of people in skilled trades occupations, caring, sales and process plant/machine operations. Some people who worked outside the home in the current study were thus likely to be in lower paid jobs and, in turn, ample evidence connects low socioeconomic status with CVD risk factors such as smoking, high body mass index and some types of cholesterol (Hamad et al., 2020; Winkleby et al., 1992). However, our SEM findings suggested that the high-risk group comprised of people who combined working both at home and outside the home during the pandemic, likely because they might have worked in sectors which were not eligible for free childcare, making them juggle additional stressors. In contrast, the UK government kept nurseries and schools open for employees in key sectors (e.g., health, food and manufacturing) who were more likely to have worked outside the home full-time. Lack of access to such support might explain the higher rates of depression and smoking found by our SEM results among those who worked both within and outside the home. Therefore, our study adds to literature showing the broader relevance of the type of job someone does to their cardiovascular health (e.g., risk of heart attacks, stroke and angina) either directly or through mediating factors such as smoking and depression.

### Limitations and future research

One limitation was that this study is not a longitudinal study therefore we cannot infer causality. Future studies should conduct long-term research that will enable inferences about causality by tracking the effects of primary/secondary risk factors at time-1 on CVDs at time-2. The survey asked participants about their history of CVDs therefore we could not calculate the incidence of heart attacks, stroke or angina after the COVID-19 pandemic started. In this study, the frequency of drinking sugary drinks, eating confectionary and changes in alcohol consumption and smoking were measured with single-item self-report measures but future studies should use objective data (where possible), such as medical records about blood sugar/alcohol levels, formal diagnoses about anxiety/depressive disorders and hospital records about CVDs. This will enable future research to longitudinally replicate our study by testing the causal effects of anxiety, depression and other variables on the incidence of future cardiovascular diseases and arrhythmia.

### Clinical implications

The results show that clinicians (e.g., cardiologists, family doctors, psychiatrists, psychologists and other mental health practitioners) should advise clients with anxiety and depression about the cardiovascular disease risks, referring clients for further screening depending on comorbidity, lifestyle, occupation and demographics. Our study also shows that clinicians should adopt a holistic approach to helping patients understand factors that impact CVD risks. They should screen for anxiety, depression, alcohol use, smoking, age, sex, occupational factors and diet as risk factors for CVDs. Clinicians working with patients for whom abnormal heart rhythm is a symptom present as part of an anxiety disorder (see e.g., Abrignani et al., 2014) should note that it could be a risk factor for CVDs. Clients with anxiety and abnormal heart rhythm should be monitored and supported through cognitive and behavioural techniques which ameliorate anxiety and thus reduce the incidence of abnormal heart rhythms. Additionally, clinicians working with clients using alcohol, smoking or a sugary diet to cope with distress should advise them about the cardiovascular risks of these maladaptive coping methods.

Clinicians should screen and follow up with clients irrespective of age because this study shows that both older and younger people have cardiovascular risks for different reasons. Clinicians should follow up with older clients who have a history of depression, and younger people with anxiety, abnormal heart rhythm and who smoke or have sugary diets because of the cardiovascular risks. Finally, clinicians should note that clients’ occupations and socio-economic status could put them at risk of poor mental health and CVDs, therefore it is important to give them advice about adaptive methods of coping with work-related stress which reduce CVD risk.
